# Stand-Up, Squat, Lunge, and Walk With a Robotic Knee and Ankle Prosthesis Under Shared Neural Control

**DOI:** 10.1109/OJEMB.2021.3104261

**Published:** 2021-08-11

**Authors:** Grace R. Hunt, Sarah Hood, Tommaso Lenzi

**Affiliations:** ^1^ Department of Mechanical Engineering and Utah Robotics CenterUniversity of Utah 7060 Salt Lake City UT 84112 USA

**Keywords:** Bionics, electromyography, robotic prosthesis, shared control, transfemoral amputee

## Abstract

Emerging robotic knee and ankle prostheses present an opportunity to restore the biomechanical function of missing biological legs, which is not possible with conventional passive prostheses. However, challenges in coordinating the robotic prosthesis movements with the user's neuromuscular system and transitioning between activities limit the real-world viability of these devices. Here we show that a shared neural control approach combining neural signals from the user's residual limb with robot control improves functional mobility in individuals with above-knee amputation. The proposed shared neural controller enables subjects to stand up and sit down under a variety of conditions, squat, lunge, walk, and seamlessly transition between activities without explicit classification of the intended movement. No other available technology can enable individuals with above-knee amputations to achieve this level of mobility. Further, we show that compared to using a conventional passive prosthesis, the proposed shared neural controller significantly reduced muscle effort in both the intact limb (21–51% decrease) and the residual limb (38–48% decrease). We also found that the body weight lifted by the prosthesis side increased significantly while standing up with the robotic leg prosthesis (49%–68% increase), leading to better loading symmetry (43–46% of body weight on the prosthesis side). By decreasing muscle effort and improving symmetry, the proposed shared neural controller has the potential to improve amputee mobility and decrease the risk of falls compared to using conventional passive prostheses.

## I. Introduction

Above-knee amputation disrupts the natural coordination of biological legs, limiting the mobility of individuals with amputations [1]. After above-knee amputation, the knee and ankle joints are replaced by passive prosthetic joints that cannot perform the biomechanical functions of the missing biological leg joints [2]. Individuals with amputations must rely on their intact leg and upper body to compensate for the limitations of the prosthesis, resulting in slower, less stable, and less efficient ambulation [3]–[5] while leading to secondary physical conditions such as back pain, osteoarthritis, and osteoporosis [1]. The limited functional mobility provided by available prostheses severely affects the quality of life of millions of individuals world-wide [6], [7]. Improved prosthesis technologies are necessary to meet the needs of this population.

Robotic prostheses present a promising solution to this problem. In contrast to conventional devices, robotic prostheses have battery-operated servomotors that can generate the torque and power necessary to imitate the biomechanical function of the missing biological leg [8]. However, appropriate controllers are necessary to synchronize the movements of the prosthesis with the user's neuromuscular system. A common approach to robotic prosthesis control is to classify the user's intended activity, such as standing up or walking [9], and then impose a pre-planned prosthesis action that imitates the behavior of an intact biological leg during the intended activity [10]–[12]. Using this approach, robotic leg prostheses have shown the ability to assist individuals with above-knee amputations in structured laboratory environments [13], [14]. However, the real world is highly variable. Timely and accurate classification of all possible variations of each ambulation activity is both challenging and critical. Any misclassification of the user's intended movement can cause the prosthesis to perform a different activity than the user expects, increasing the likelihood of falls and injuries [15]. Moreover, every activity requires a dedicated controller manually tuned for each subject [12], [16]. Addressing these challenges is essential to achieving the full potential of robotic leg prostheses.

Significant effort has been made to improve controllers for robotic leg prostheses. Using electromyography (EMG) from residual-limb muscles has been shown to improve classification accuracy, compared to using mechanical sensors alone [17]–[19]. Computer vision and range sensors have also been proposed to improve classification accuracy [20]–[22], although their real-world viability is limited by concerns with camera placement, privacy [23], and societal acceptance [24]. Regardless of the specific sensors used, training an activity classification algorithm requires the user to perform multiple repetitions of each activity as well as the transitions between activities, which can be taxing and even dangerous for the user without the supervision of trained personnel [25]. Most importantly, even with perfect classification, every possible variation of each activity requires a separate, manually tuned controller [16], [25].

Controllers based on continuous use of EMG signals have been proposed as alternatives to pre-tuned controllers based on mechanical sensors [26] with the goal of improving adaptability to real world variability. However, these controllers require subject- and session-specific training of the machine learning algorithm used to translate the EMG signals into effective commands for the prosthesis [27], [28]. Moreover, intensive, multi-week, multi-session subject training is needed to, at best, match the performance of controllers which do not use EMG [28]. Most importantly, a separate EMG controller must be used for each ambulation mode (e.g., stair ascent [27], walking [28]). Thus, robotic leg prostheses based on classifying the user's intended activity and switching between separate controllers have fundamental limitations that reduces safety and usability in the real world.

In this paper, we show an alternative control approach for robotic knee and ankle prostheses. We propose a shared neural control approach that combines neural signals and robot control to enable users to perform multiple ambulation activities and eliminates the need for subject training and machine training. Rather than explicitly classifying the user's intended ambulation activity and enforcing a pre-planned prostheses action, we provide users with continuous volitional control of a robotic knee and ankle prosthesis using EMG. The proposed shared neural controller enabled two individuals with above-knee amputations to stand up, sit down, squat, lunge, walk, and seamlessly transition between activities without explicitly classifying the ambulation mode intended by the user or enforcing pre-planned prostheses actions. No other available technology can enable individuals with above-knee amputations to achieve this level of mobility. A prosthesis controller with this functionality has the potential to improve the mobility of individuals with above-knee amputation in the real world.

## II. Results

**Figure 1.**
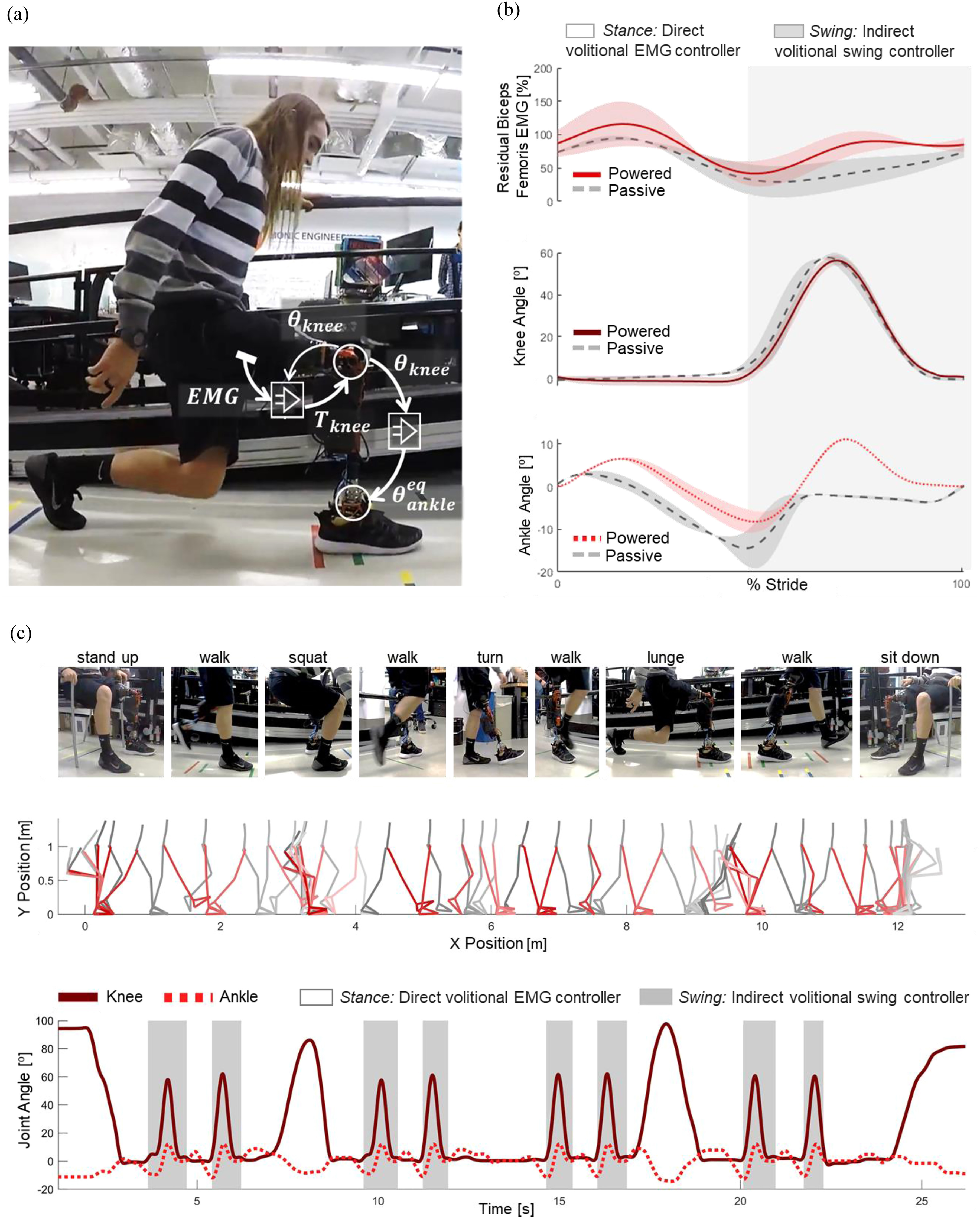
Walking and transitioning between movements with shared neural controller. (a) Subject S1 lunges with the proposed shared controller. Overlay: Diagram of robotic knee-ankle prosthesis and proposed shared neural controller. $EM{G_{BF}}$, the EMG signal from the residual biceps femoris, controls ${T_{knee}}$, the knee extension torque provided by the prosthesis. ${T_{knee}}$ determines ${\theta _{knee}}$, the angle of the prosthesis knee, which then controls ${\theta _{ankle}}$, the angle of the prosthesis ankle. (b) Comparison of walking using a robotic knee-ankle prosthesis with the proposed shared neural controller (red, solid line), compared to walking with passive prostheses (grey, dashed line). Bolded lines indicate between-subject means, and shaded areas indicate between-subject standard errors. The *Swing* phase of walk, when an indirect volitional swing controller controlled the prosthesis movement, is shaded in grey. The *Stance* phase of walk, when the prosthesis was controlled using EMG, is not shaded. (c) Ambulation circuit demonstrating seamless transition between walking and various activities for subject S1. The subject stands up, walks, squats, walks, turns, walks, lunges, walks, and sits down. Still-frames from video are shown, as well as cartesian positions of body segments during the circuit. Note that the still-frames and cartesian coordinates during the second half of the circuit (after the subject turned around), have been mirrored. The graph shows the knee and ankle angle during this circuit of activities. The *Swing* phase of walk, when an indirect volitional swing controller controlled the prosthesis movement, is shaded in grey. *Stance*, when the prosthesis was controlled using EMG, is not shaded.

A single surface EMG electrode (13E202=60, Ottobock) is placed on the posterior side of the residual limbs of each subject to measure the activation of a residual hamstring muscle—the biceps femoris (Fig. 1(a)). After above-knee amputation, the biceps femoris loses its ability to flex the knee and becomes a monoarticular hip extensor [29]. The EMG signal of the biceps femoris is translated into a desired knee extension torque using a position-dependent gain, so that higher knee flexion angles result in a higher level of torque for the same muscle activation. The equilibrium angle of the robotic ankle joint is defined as a linear function of the prosthetic knee position (Fig. 1(a)), mimicking the physiological knee-ankle relationship observed in non-amputee individuals during standing up [30]. The proposed shared neural controller was implemented in a robotic knee and ankle prosthesis (Fig. 1(a), Supplementary Fig. 1) and tested by two individuals with above-knee amputations, who performed sit-to-stand, stand-to-sit, squats, lunges, level-ground walking, and transitions between activities.

Subjects walked on level ground using both their prescribed passive prosthesis and the robotic prosthesis with the proposed shared neural controller. The residual biceps femoris EMG activations were highest during the stance phase of walk (0 50% stride) and had similar patterns and peaks during walking with passive and robotic prostheses (Fig. 1(b)). For both subjects, neither the magnitude nor peak of the residual biceps femoris EMG activations were significantly different between walking with the robotic and passive prostheses (peak magnitude: p = 0.012 for S1, p = 0.066 for S2, peak timing: p = 0.219 for S1, p = 0.179 for S2, Supplementary Table 2). With the shared neural controller, the EMG activation in *Stance* resulted in knee extension torque (see Methods) which prevented the knee joint to collapse while the subject weighted it during stance (Fig. 1(b)). Because the knee was fully extended during *Stance*, the desired ankle angle remained neutral, while the impedance control allowed the ankle's measured angle to change from plantarflexion (positive) to dorsiflexion (negative) (Fig. 1(b)), imitating the intact biological ankle kinematics [30]. Thus, walking with the shared neural controller did not require significant alterations of the residual biceps femoris EMG activations.

Subjects completed an ambulation circuit in which they stood up, took two steps, squatted, took two steps, turned, took two steps, lunged, took another two steps, turned, and sat down. A video of the tests is available in the Supplementary materials (Video_03_ambulation_circuit). During the ambulation circuit, the robotic prosthesis seamlessly switched between two different states—*Stance* and *Swing.* For all ambulation activities, the actions of the robotic knee and ankle prosthesis during *Stance* (un-shaded areas in Fig. 1(c)) were controlled by the subject's residual biceps femoris EMG using the proposed shared neural controller (Fig. 1(a)), whereas the actions of the robotic knee and ankle during *Swing* (shaded areas in Fig. 1(c)) were controlled using an indirect volitional controller [31]. For both subjects, the ankle and knee angle trajectories did not show discontinuities at the transitions between different activities or controller states (Fig. 1(c)). The knee kinematics showed noticeable differences during the different activities in the ambulation circuit. The knee angle peaked at 86° in squat, 98° in lunge, and 60.5 ± 0.62° during walk for S1, and 88° in squat, 68° in lunge, and 56.46 ± 0.72° during walk for S2 (see Supplementary Fig. 3). Subjects were able to seamlessly transition between walking and other activities with the robotic prosthesis with the shared neural controller.

**Figure 2.**
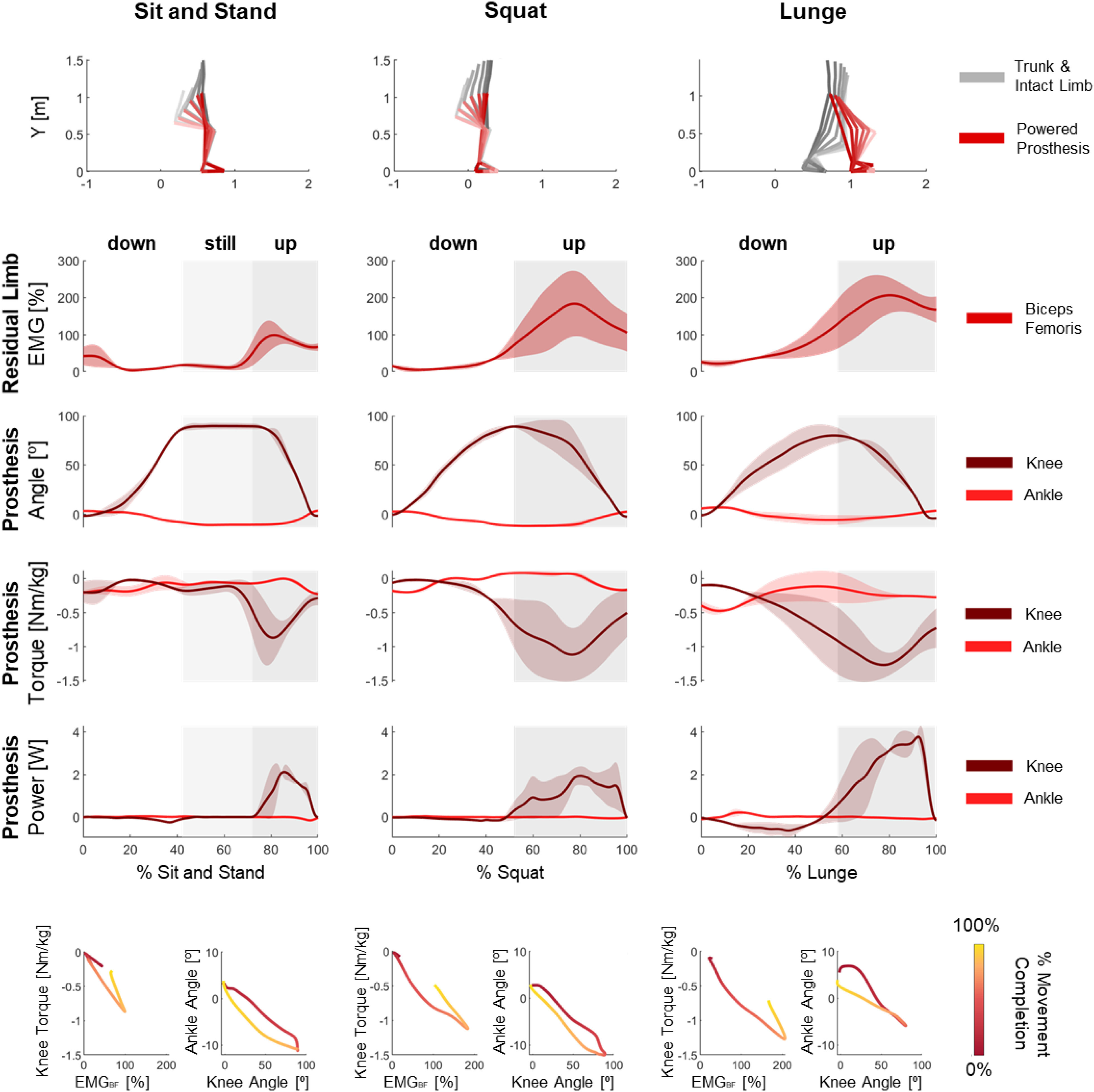
Sit and stand, squat, and lunge with the robotic prosthesis under shared neural control. Top row: Body segment positions in cartesian space during each movement. Rows 2-5: Time-series of residual limb biceps femoris EMG signals and the joint angles, joint torques, and joint powers at the prosthesis knee (dark red) and prosthesis ankle joint (light red) during each activity. Shading indicates parts of the movement: during sit and stand, the subjects sat down (white), sat still in the chair (light grey shading), and then stood up (dark grey shading); during squat and lunge, the subjects moved down into a squat or lunge (no shading) and then moved back up to standing (grey shading). Bolded lines indicate between-subject means, and shaded areas indicate between-subject standard errors. Bottom row: Between-subject mean of prosthesis knee torque vs. residual limb biceps femoris EMG, and prosthesis ankle angle vs. prosthesis knee angle. Gradient indicates movement completion: values at the beginning of each movement are red, and values at the end of each movement are yellow. Supplementary Figures 4 and 5 show these results of this test for S1 and S2, respectively. .

Subjects performed sit-to-stand, squat, and lunge with the robotic prosthesis using the proposed shared neural controller (Video_01_sts_squat_lunge). The average prosthesis knee and ankle joint angles had similar ranges of motion during the three activities (Fig. 2, Supplementary Table 3). In contrast, the residual biceps femoris EMG activation and the prosthesis knee power showed significant differences between activities for both subjects (Fig. 2, p < 0.01, Supplementary Table 3). Average residual biceps femoris EMG activations peaked at 99 ± 42 %, 184 ± 108 %, and 206 ± 59 %, for sit and stand, squat, and lunge respectively (Fig. 2, Supplementary Table 3). Average knee power peaked at 2.12 ± 0.31 W during stand-up, at 1.94 ± 0.44 W during squat, and at 3.77 ± 0.30 W during lunge (Fig. 2, Supplementary Table 3). There were visible differences in knee extension torque, which peaked at 0.87 ± 0.35 Nm/kg, 1.12 ± 0.39 Nm/kg, and 1.27 ± 0.19 Nm/kg for sit and stand, squat, and lunge, respectively (Fig. 2, p = 0.01 for S1, p < 0.01 for S2, Supplementary Table 3). Lunge was the fastest activity, with an average knee angular velocity peaking at 149.7 ± 43.6 °/sec, compared to 106.2 ± 20.2 °/sec for squat and 134.3 ± 3.8 °/sec for sit and stand. The torque and power at the ankle joint were consistently smaller than at the knee joint for all the activities (Fig. 2, Supplementary Table 3). The analysis of the knee torque as a function of the EMG activation shows a non-linear, non-monotonic relationship, where the same knee torque can result from different EMG activations (Fig. 2), a result of the position-dependent gain used to compute the desired knee torque. Although the equilibrium angle of the ankle changes linearly with the measured knee angle, the relationship between the measured ankle angle and the measured knee angle shows an elongated circular shape (Fig. 2), a result of the low-level impedance control used for the ankle. The proposed shared neural controller enabled the subjects to adapt the prosthesis movements as necessary to perform sit-to-stand, squats, and lunges with the same controller.

**Figure 3.**
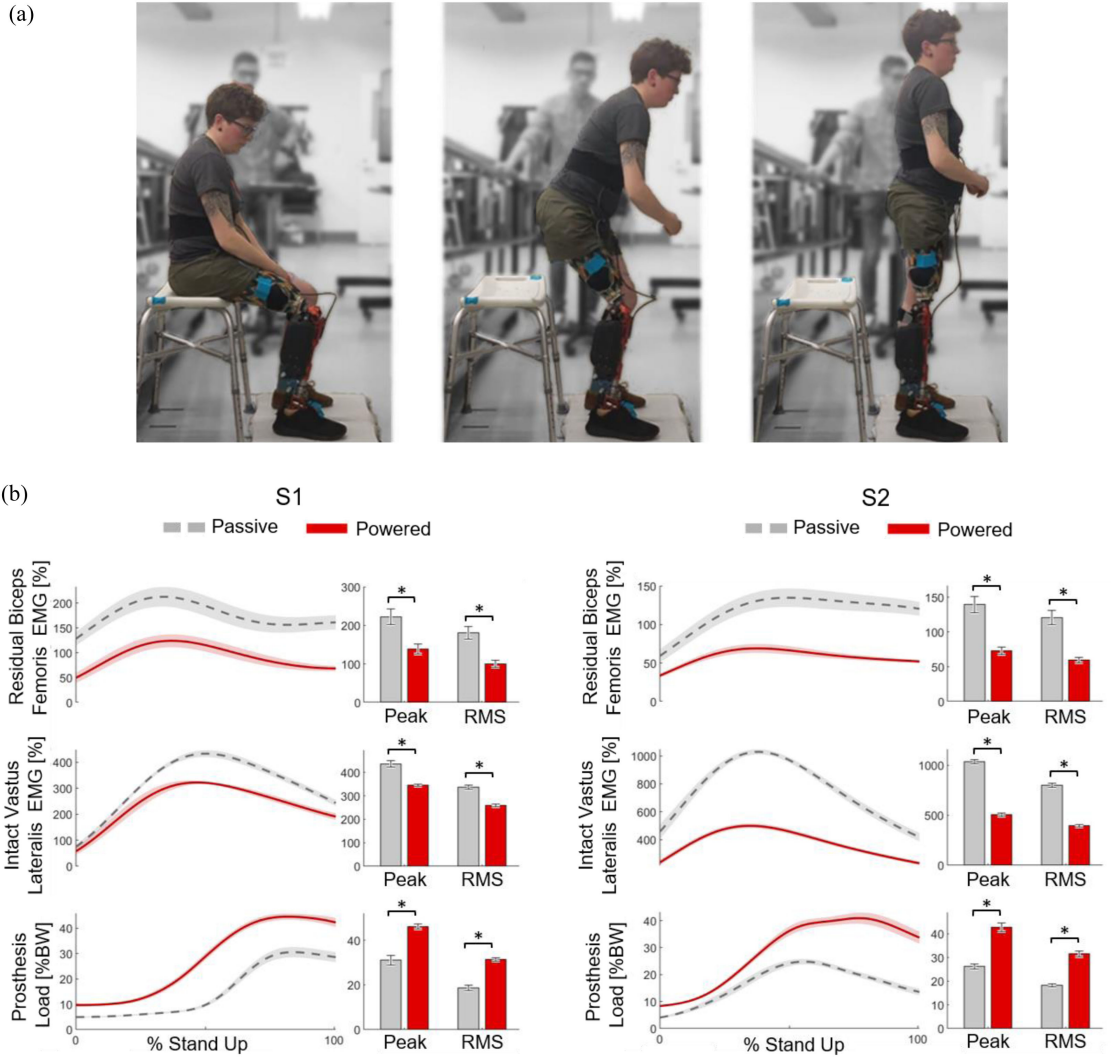
Standing up with the proposed shared neural controller compared with standard of care. (a) Subject S2 stands up with robotic knee-ankle prosthesis using the proposed shared neural controller. (b) Comparison between stand-up with robotic prosthesis under proposed shared neural controller (red) vs. standard of care (passive prosthesis) (grey) for each subject. In line plots, bolded lines indicate within-subject means, and shaded areas indicate within-subject standard errors. Bar plots show within-subject means (bar) and standard errors (brackets). Asterisks indicate statistically significant differences between prosthesis conditions.

Subjects performed sit-to-stand with a robotic prosthesis using the proposed shared neural controller (Fig. 3(a)) and with their prescribed passive prostheses while we measured the EMG activations of the residual limb biceps femoris and the intact limb vastus lateralis muscles, as well as ground reaction forces from both limbs. For both subjects and both muscles, the EMG activations were significantly lower with the robotic prosthesis than the passive prosthesis (Fig. 3(b), p < 0.01, Supplementary Table 4). With the robotic prosthesis, the peak of the residual biceps femoris EMG was reduced by 38% for S1 and by 48% for S2 (Fig. 3(b)), and the RMS was reduced by 45% for S1 and 50% for S2 (Fig. 3(b)). Peak intact vastus lateralis EMG was reduced by 21% for S1 and 51% for S2 (Fig. 3(b)), and the RMS was reduced by 23% for S1 and 51% for S2 (Fig. 3(b)). The EMG activations of both the residual biceps femoris and intact vastus lateralis muscle during passive and robotic stand-up had different magnitudes but similar patterns, with no significant difference in the timing of the peak activation between conditions (Fig. 3(b), p = 0.83 for S1 and p = 0.94 for S2, Supplementary Table 4). The robotic prosthesis lifted significantly more of the subjects’ weight during stand-up compared to the passive prostheses (Fig. 3(b), p < 0.01, Supplementary Table 4). With the robotic prosthesis, the peak of the load lifted by the prosthesis increased by 49% for S1 and 63% for S2 (Fig. 3(b)), whereas the RMS of the load on the prosthesis side increased 68% for S1 and 73% for S2 (Fig. 3(b)). The resulting peak loading on the prosthesis side was 46.1 ± 4.29 % of body weight for S1 and 42.7 ± 6.98 % of body weight for S2 during robotic stand-up. Thus, the robotic prosthesis with the proposed shared neural controller significantly reduced muscle effort and improved symmetry during standing up, compared to standing up with conventional passive prostheses.

Subjects performed a series of sit-to-stands under different conditions that could be encountered in real life with the robotic prosthesis under shared neural control (Supplementary Fig. 6, Video_02_different_sts). When asked to stand up as quickly and as slowly as possible, subjects were to stand up from chairs of different heights, ranging from a minimum of 38 cm (the height of a standard toilet) to a maximum of 54 cm (the height of a tall chair). When subjects stood up from a shorter chair, their EMG activations were significantly higher (Supplementary Fig. 6, p < 0.01, Supplementary Table 5). Subjects were also able to stand up while wearing a 30-lb backpack, which resulted in significantly larger prosthesis knee torques and EMG activations compared to standing up without the backpack (Supplementary Fig. 6, p < 0.01, Supplementary Table 5). Subjects were also able to stand-up partially, as if they had begun standing up and then changed their mind. Compared to normal stand-up, both subjects’ knee range of motion decreased significantly during partial stand-up, from 94° to 44° for S1 and from 92° to 49° for S2 (Supplementary Fig. 6, p < 0.01, Supplementary Table 5). Thus, the proposed shared neural controller enabled the subjects to change the prosthesis movement as necessary to stand up with different timing, geometry, and loading conditions.

## III. Discussion

Robotic leg prostheses can actively generate torque and power as necessary to imitate the biomechanical functions of the missing biological leg [8]. Proper synchronization of the robotic leg prosthesis with the user's neuromuscular system is necessary to achieve this goal. Synchronization is particularly challenging in the real world, due to the high variability of the environment and the prosthesis users. With the proposed shared neural controller, a user can voluntarily change the torque generated by the robotic knee prosthesis, controlling the timing and amount of energy provided by the robotic prosthesis. As a result, the robotic leg prosthesis significantly reduces the amount of compensatory work done by the user's intact and residual limb. Compared to using a conventional passive prosthesis, the proposed shared neural controller significantly reduced muscle effort in both the intact limb (21–51% decrease) and the residual limb (38–48% decrease) (Fig. 3). We also found that the body weight lifted by the prosthesis side increased significantly while standing up with the robotic leg prosthesis (49%–68% increase), leading to better loading symmetry (43–46% of body weight on the prosthesis side) (Fig. 3). Decreased muscle effort and increased prosthesis loading are clinically meaningful because muscle fatigue and loading asymmetry have been linked to increased fall risk [32], [33]. The proposed shared neural controller allowed for substantial variations in both activity and environment, enabling users to stand up from different chairs (38–54 cm), stand up slower and faster (0.5–2.2 sec), stand up while carrying a load (0–30 lbs.), as well as squat, lunge, and walk. Most importantly, subjects were also able to seamlessly transition between activities, which is critical for ambulation in the real world.

Robotic prosthesis controllers commonly aim to identify the user's intended activity [9], [10] and impose a pre-planned prosthesis action that imitates the movement of biological limbs during that activity [11]–[14]. There are considerable limitations to the real-world viability of this approach. The classification algorithms used to identify the subject's intended activity must be trained using subject-specific, labelled data [10]. The classification accuracy decreases over time [34] and retraining can be difficult and unsafe for the user to perform at home. Even a single misclassification can cause a misstep, resulting in a fall and, potentially, an injury [15]. Most importantly, every activity and every variation of an activity requires a dedicated controller, and each of these controllers must be trained or manually tuned for each subject [25], which is time consuming and requires expertise not commonly available to clinicians. Our study presents a fundamental departure from this paradigm. Rather than aiming to improve classification of the user's intended activity, we aim to give the user volitional control over the robotic prosthesis using neural commands from their residual limb.

Using EMG signals from antagonist muscle groups works well for repositioning the robotic leg when the subject is sitting [35]–[38] but not for ambulation. Previous studies have demonstrated direct antagonist EMG control for specific ambulation activities, such as stair ascent [27] and walking [28]. However, co-activation of the residual-limb muscles was shown to be a key limitation to the viability of this antagonist approach for weight bearing activities [27], [28]. These direct EMG controllers can, at best, match the performance of non-neural controllers and require extensive controller training, subject-specific tuning, and intensive, multi-week, multi-session subject training. Moreover, they are limited to one specific ambulation activity [28]. Thus, previous EMG-based controllers still require classification of the intended ambulation activity, reducing their clinical viability. Surgical interventions that fuse the antagonist muscle pairs [39] may alleviate the co-activation problem by restoring proprioception [40], but these interventions have never been attempted with above-knee amputee patients. Here we show that a shared neural controller that combines neural signals from a single hip extensor muscle with robot control enables standing up, squatting, lunging, and walking without explicit classification of the user's intended activity, controller tuning, or subject training.

In non-amputee individuals, knee extension torque is provided by the quadriceps muscle. Thus, it may seem logical to use the EMG signals produced by a quadricep muscle to drive the knee extension torque generated by a robotic prosthesis. However, after above-knee amputation, the quadricep muscles lose their knee extension function. The vastus muscles atrophy [41] and the rectus femoris becomes a monoarticular hip flexor. In this study, we show that the biceps femoris, a biarticular hamstring muscle in nonamputee individuals, provides a viable alternative to drive the knee extension torque generated by a robotic prosthesis. After above-knee amputation, the biceps femoris loses its knee flexion function, but its hip extension function is retained. The biceps femoris is naturally active during standing up (Fig. 3), when both hip extension torque and knee extension torque are required to counteract gravity. The biceps femoris is also naturally active during the stance phase of walking (Fig. 1), when hip extension torque is necessary to propel the body forward and upward and knee extension torque is necessary to prevent the knee from collapsing. Because the biceps femoris naturally activates when knee extension torque is necessary, such as during standing up (Fig. 3) and stance phase of walking (Fig. 1), users do not need to learn a new muscle activation pattern to use the proposed shared neural controller. When knee flexion torque is required, as in the swing phase of walking, we use robot control in the form of an indirect volitional control that automatically adapts the prosthesis trajectory based on the movements of the user's residual limb [31], enabling users to modulate the foot clearance while walking and crossing over obstacles without explicit classification of the environment. Thus, the EMG signal from the biceps femoris provides an intuitive input to provide direct volitional control of the robotic prosthesis during movements that require knee extension torque, and the robot control provides indirect volitional control during movements that require knee flexion torque.

After above-knee amputation, all muscles that control the ankle joint are removed and EMG signals from the muscles of the residual limb do not provide an intuitive way to control the prosthetic ankle. Surgical interventions, such as targeted muscle reinnervation [42], [43], and peripheral nerve interfaces [44] have the potential to provide signals for intuitive control of the prosthesis ankle joint. However, these techniques have not shown the ability to directly control a robotic prosthesis during walking, standing up, or other activities. To address this limitation, we combined neural signals and robot control. In non-amputee individuals, the ankle and knee move in synchrony during standing-up movements, and knee extension is mirrored by ankle plantarflexion. Our controller captures this natural coordination with a linear relationship controlling the equilibrium angle of the prosthesis ankle joint as a function of the measured prosthesis knee angle. This control approach enables the prosthetic foot to lay flat on the ground and support users while they perform many different activities, including standing up, sitting down, squatting, and lunging. The virtual impedance of the ankle joint adds the flexibility necessary to walk in addition to performing standing up activities, without any tuning or calibration of the controller. Because the prosthesis knee position depends on the EMG-controlled prosthesis knee torque, this shared control strategy provides users with indirect volitional control of the prosthesis ankle joint.

This study is limited by the small number of subjects enrolled. It is common in the field of wearable robotics to initially test new technologies with a small number of subjects [44], [45]. Power analysis based on observed effect size and variability will be used to determine the appropriate number of subjects for a future clinical study. Another important limitation is that both study participants are young, active individuals who underwent a traumatic amputation. Further investigation is necessary to address the viability of the proposed shared neural controller for use by older and less active individuals, who are more commonly part of the dysvascular amputee population. Although subjects reported that the shared neural controller was easy to use and did not require mental strain or attention, which is an known issue in other neural controllers [46], further studies are necessary to assess the attentional requirements [47]. Future work will focus on extending the proposed shared neural controller to include more ambulation activities such as stair climbing and assessing mobility in a broader population.

## IV. Conclusion

Robotic leg prostheses promise to improve the ambulation ability of millions of individuals with lower-limb amputations. Effective, intuitive, and safe controllers are essential to achieve this goal. By putting the user in control of the robotic leg prosthesis, the proposed shared neural controller enables standing up under a variety of conditions, squatting, lunging, walking, and seamlessly transitioning between activities—none of which are possible with conventional passive prostheses or other robotic prosthesis controllers. Both subjects were able to perform all activities without training, specific instruction, failed attempts, or visual feedback. No subject-specific tuning of the controller was necessary other than adjusting the gain of the EMG sensor as recommended by the manufacturer. Compared to conventional passive prostheses, we show significant improvements in weight bearing symmetry and muscle effort. The proposed robotic leg prosthesis controller has the potential to improve the mobility of individuals with above-knee amputation in the real world.

## V. Materials and Methods

### A. Shared Neural Control

At the high-level, we use a finite state-machine after [48]. This finite-state machine comprises two different states—*Stance* and *Swing*. When, the prosthesis contacts the ground, as detected by the ground reaction force exceeding 50 N [49], the finite-state machine enters *Stance*. From *Stance*, the finite-state machine transitions to *Swing* if the shank position and shank velocity are below thresholds while the knee position is below a threshold. The parameters for the finite-state machine are fixed and do not need to be tuned for different users [48]. In *Swing,* we use an indirect volitional controller that was previously tested with individuals with above-knee amputation [31], [48], [50], [51]. In *Stance*, we use a direct volitional controller based on EMG from the residual limb. The proposed *Stance* controller is used for standing up, sitting down, squatting, lunging, quiet standing, as well as for the *Stance* phase of walking. *Stance* phase begins with prosthesis heel-strike and ends with sound side heel-strike, at which point the finite-state machine transitions to *Swing*, during which the prosthesis is controlled using an indirect volitional controller [31].

Two different low-level controllers are used in *Stance* for the knee and the ankle joint. The knee joint extension torque is controlled using proportional EMG control using the following equation:

\begin{equation*}
\left\{ \begin{array}{l} {T_{knee}^{des} = \frac{{EMG}}{{EM{G_{max}}}}{\rm{\ }}G{\rm{\ }}}\\ {G = {G_0} + {G_1}{\rm{\ }}{\theta _{knee}}{\rm{\ \ \ }}} \end{array}\right. \tag{1}
\end{equation*}

The EMG signal from the biceps femoris ($EMG$) is normalized using its average peak recorded during walking with passive prosthesis ($EM{G_{max}}$) multiplied by a position-dependent gain ($G$) to obtain the desired knee torque ($T_{knee}^{des}$). The position-dependent gain is calculated using a linear curve with an offset (${G_0} = 30^\circ $) following (1). Because the multiplication factor $({G_1}$ = 0.625) is positive, the EMG gain ($G$) increases with the knee angle position ($\ {\theta _{knee}}$), resulting in higher sensitivity of the desired torque to the EMG signal for more flexed knee joint angles.

The ankle joint is controlled using an impedance-based control strategy [52] with fixed stiffness and damping and variable equilibrium position ($\theta _{ankle}^{eq}$). The ankle equilibrium position ($\theta _{ankle}^{eq}$) changes as a function of the measured knee position ($\ {\theta _{knee}}$) based on the linear relationship determined by the following equation where *k* = −0.133.

\begin{equation*}
\left \{ \begin{array}{l} {\theta _{ankle}^{eq} = k\ {\theta _{knee}}\ \ \ \ \ \ \ \ \ \ \ \ \ \ \ \ \ \ \forall {\theta _{knee}} \geq 0\ }\\ {\theta _{ankle}^{eq} = 0\ \ \ \ \ \ \ \ \ \ \ \ \ \ \ \ \ \ \ \ \ \ \ \ \ \ \ \ \ \ \forall {\theta _{knee}} < 0} \end{array} \right. \tag{2}
\end{equation*}

When the knee is fully extended ($\theta _{knee}^{} = 0$), the ankle equilibrium position is set to a neutral standing position ($\theta _{ankle}^{eq} = 0$). When the knee flexes ($\dot{\theta }_{knee}^{} > 0$), the ankle dorsiflexes ($\dot{\theta }_{des}^{eq} < 0$). As shown in (2), the equilibrium angle of the ankle ($\theta _{ankle}^{eq}$) reaches a maximum of 12° when the knee joint is flexed at 90°. Also, as shown in. (2), the ankle equilibrium position is never positive, so the ankle joint does not actively plantarflex in *Stance*. Notably, this relationship does not limit the ability of the powered ankle to actively plantarflex in late stance during walking. The powered leg transitions to the *Swing* controller roughly at sound side heel-strike (i.e., beginning of double stance phase). The *Swing* controller has a pre-swing state that enables the powered prosthesis to provide active push off, providing net-positive energy injection into the gait cycle proportional to walking speed, as demonstrated in our previous work [48].

### B. Participant Information

Two subjects with above-knee amputations were recruited for this study. Inclusion criteria were unilateral above-knee amputation, ability to walk without assistance, ability to stand from a chair without assistance, and daily use of prescribed prosthesis. Exclusion criteria included any musculoskeletal, cardiovascular, neurological, or other impairments that would prevent a subject from completing the study activities. More details on the study participants can be found in Table I. The study protocol was approved by the Institutional Review Board of the University of Utah (Protocol Number IRB_00103197, Approval Date 7/21/2017). Before the experiment started, subjects provided written informed consent and written permission to publish photographs and videos of the experiments. A certified prosthetist was present during all experiments. Both subjects had previous experience using the robotic knee and ankle prosthesis used in this study. However, neither subject had previous experience using the proposed shared neural controller.

### C. Experimental Protocol

The purpose of this study was to test the functionality of a shared neural controller for robotic knee-ankle prostheses. A series of tests were performed by the subjects using their prescribed passive prosthesis and our robotic knee and ankle prosthesis under shared neural control. Both subjects performed the tests with their prescribed passive prosthesis first. Details about the number of repetitions of each activity that were recorded and used are specified below.

*Walking****:*** Subjects were asked to walk on level ground at their preferred speed and cadence. The 24-foot walkway allowed for 4-5 consecutive strides. Subjects walked back and forth until at least 20 steady-state strides were recorded (excluding first, last, and turning steps). Both subjects performed this walking test with their prescribed passive prosthesis and the robotic prosthesis with the proposed shared neural controller.

**TABLE I:** Participant Information

Subject identifier	S1	S2
Age (years)	26	30
Height (m)	1.78	1.60
Weight (kg)	64.9	59.0
Amputation side	Right	Left
Amputation cause	Trauma	Trauma
Amputation years	5	10

*Sit-to-Stand****:***Subjects were asked to stand up and sit down from an armless, adjustable-height chair, with each foot placed on a separate force plate (Video_01_sts_squat_lunge). For all sit-to-stand transitions, subjects were asked to position their feet evenly on the force plates. Subjects were asked not to touch the chair with their hands, so they could not use their hands to push themselves up. However, subjects were allowed to place their hands on their thighs if needed. Subject performed sit-to-stand transitions at their comfortable speed with their prescribed passive prostheses using a standard chair height of 50 cm (measured from the top of the force plates to the top of the chair seat). Next, subjects performed sit-to-stand transitions with the robotic prosthesis under different conditions, selected to simulate real-world conditions (Video_02_different_sts). Subjects were asked to perform sit-to-stand transitions with three different chair heights—tall, standard, short. The standard chair height was 50 cm—the same used for the prescribed passive prosthesis. The tall chair height was 54 cm, which is between the height of a dining chair and a counter chair [53]. The height of the short chair was 42 cm for S1 and 38 cm for S2. This height is similar to that of a standard toilet [54]. The difference in height between subjects was due to the range of motion of the robotic prosthesis, which was limited by the user's socket for S1. Subjects were also asked to perform partial sit-to-stand transfers using the armless chair set at standard height (50 cm). Specifically, they were asked to stand up partway and immediately sit down again, as if they had begun standing up but changed their mind and returned to a seated position. Finally, subjects were asked to stand up and sit down from standard-height chair (50 cm) as quickly as possible and as slowly as possible. Subjects performed 5–10 repetitions of each variation, and the last 5 were used.

*Squat****:***Subjects were asked to squat with the robotic prosthesis while holding onto a handrail for safety (Video_01_sts_squat_lunge). Subjects were encouraged to squat as deep as they felt confident and safe. Subjects performed 10–12 repetitions, and the last 6 were used.

*Lunge****:*** Subjects were asked to lunge with the robotic prosthesis in front, while holding onto a side handrail for safety (Video_01_sts_squat_lunge). Subjects were asked to place the prosthesis in front of them and bend both knees to lunge as deep as they felt comfortable, and then to step through the lunge and take a step before performing another lunge. Subjects performed 10-12 repetitions, and the last 6 were used.

*Ambulation Circuit****:*** Subjects were asked to complete an ambulation circuit with different activities connected by walking (Video_03_ambulation_circuit). The circuit proceeded as follows: stand up from a chair, take two steps, squat/lunge, take two steps, turn, take two steps, lunge/squat, take another two steps, turn, and sit down into a chair. S1 subject performed the lunge first and the squat second, and S2 performed the squat first and the lunge second. The entire circuit was performed next to a handrail, and the subjects were told to hold onto it if they felt it was necessary. A chair with arms was used, and subjects were not given specific instructions about whether to use their hands to stand up and sit down.

## Supplementary Materials

Supplementary Materials PDF: Supplementary information about the Utah Lightweight Leg, additional details about experimental protocol, figure with subject S2’s ambulation circuit, tables with additional statistical analyses of data from figures, and figure with variations of sit-to-stand.

**Supplementary Video 1: Sit-to-stand, squat, and lunge.** Subject S1 performs sit-to-stand, stand-to-sit, squat, and lunge using the robotic prosthesis under the proposed shared neural controller.

**Supplementary Video 2: Variations of sit-to-stand.** Subject S2 performs variations of sit-to-stand movement, demonstrating the robustness of the proposed neural controller to real-world variability. Subject performs sit-to-stand fast, slow, partially, from a tall chair, from a short chair, and while wearing a backpack which weighs 30lbs.

**Supplementary Video 3: Ambulation circuit.** Subject S1 performs ambulation circuit, in which he stands up from chair, takes two steps, squats, takes two steps, turns around, takes two steps, lunges, takes two steps, and sits down in a chair.
